# Open decortication for patients presenting with stage III tuberculous empyema with low density lines on CT imaging

**DOI:** 10.1038/s41598-023-36420-w

**Published:** 2023-06-14

**Authors:** Xiaoyu Liu, Xuan Wang, Jian Sheng, Yuhui Jiang, Li Li, Xiyong Dai

**Affiliations:** Wuhan Pulmonary Hospital, Wuhan Institute for Tuberculosis Control, Wuhan, 430030 Hubei China

**Keywords:** Diseases, Medical research, Pathogenesis

## Abstract

To explore the influence of CT findings on the optimal timing of open decorticationin patients with stage III tuberculous empyema. A total of 80 patients with stage III tuberculous empyema who had undergone open decortications were recruited; 44 patients had chest CT findings indicating low-density lines, while 36 patients did not show this imaging finding. Demographic data, perioperative data and preoperative and postoperative chest CT images were collected. In the low-density line group, the duration of disease (*P* = 0.0030) and the preoperative anti-tuberculosis time (*P* = 0.0016) were longer than those of the group without low-density lines, and the ESR (*P* = 0.0218), CRP (*P* = 0.0027) and leukocyte count (*P* = 0.0339) were lower in the low-density line group. Additionally, in the median operative time (*P* = 0.0003), intraoperative blood loss (*P* < 0.0001), volume of catheter drainage during 48 h after operation (*P* = 0.0067), chest tube duration (*P* < 0.0001), and length of hospital stay (*P* = 0.0154) were significantly lower in the low-density line group than in the group without low-density lines. A total of 88.64% of participants in the low-density line group showed hyperplasia with hyaline degeneration in pathological examination, which was observed only in 41.67% of patients without low-density lines. In addition, gaseous necrosis was considerably higher in patients without a low-density line (*P* = 0.004), while the low-density line group had a higher rate of treatment success (*P* < 0.05). Patients with stage III tuberculous empyema presenting with low-density lines around the thickened fibrous pleural rind on preoperative CT imaging may be good candidates for open decortication.

## Introduction

Pleural empyema is an infectious disease of the pleural space that accompanies bacterial pneumonia^1^. Tuberculous empyema develops due to the rupture of pulmonary lesions or the dissemination of primary pulmonary disease through lymph or blood, and such a development covers three stages: stage I is an exudative phase; stage II represents a fibrinopurulent phase; and stage III refers to an organizing or consolidation phase^2,3^. Surgical management has become an important treatment option for patients with tuberculous empyema, and surgical methods are closely related to disease progression^3^. Chronic fibrous pleural rind, which covers the visceral pleura and parietal pleura, is a unique feature of stage III tuberculous empyema^4^, which is usually treated by removing the thickened visceral and parietal fibrous pleura using the traditional method of surgical open decortication, especially in developing countries^5,6^. The thickened fiberboard covers the purulent pleural fluid, forming an encapsulated empyema, which can result in atelectasis. In clinical practice, the surgical opportunity is mainly determined by the course of disease and the time of preoperative anti-tuberculous treatment. However, tuberculosis presents a variety of different pathological manifestations in the thoracic cavity. Even the conditions and postoperative complications in the thoracic cavity of all stage III patients are not completely the same, thereby making it difficult to determine reference values of traditional pathological staging. Additionally, the surgical treatment of tuberculous empyema is still challenging.

In recent years, at our institution, preoperative thoracic computed tomography (CT) scans have revealed that some patients with a low-density line between the parietal fibrous pleural rind and chest wall have better efficacy than those without this finding on CT scan while performing open decortication. Therefore, the present study aimed to explore the differences inpre, intra, and postoperative characteristics between the two groups.

## Materials and methods

All methods and procedures were carried out in accordance with the principles contained in the Declaration of Helsinki.

### Participants and data collection

This study was approved by the Ethical Committee of the Wuhan Pulmonary Hospital, Ethics No: (2020)46. Data were collected from patients with stage III tuberculous empyema who underwent open decortication in the Thoracic Surgery Department of Wuhan Pulmonary Hospital from January 2015 to January 2019. The inclusion criteria were as follows: (i) positive for acid-fast bacilli stain or positive by mycobacterium culture, PCR, or GeneXpert MTB/RIF Ultra assay confirmation of the disease; (ii) free of drug-resistant tuberculosis; and (iii) treated according to a 4-drug (including isoniazid, rifampicin, pyrazinamide, and ethambutol) anti-tuberculous regimen before the surgical intervention. The exclusion criteria were as follows: (i) complicated with diabetes or HIV infection or other diseases involving the immune system or (ii) subject to other surgical methods at the same time. A total of 80 patients were ultimately enrolled and categorized into two groups according to chest CT features: the low-density line group included 44 patients with a clearly visible gap between the parietal pleural rind and the chest wall; and the group without low-density lines included 36 patients without such a finding.

The following patient data were collected and analysed in each group: duration of disease, treatment duration with anti-tuberculosis drugs, duration of surgery, intraoperative blood loss during operation, time for carrying the drainage tube, cure rate operation, pathological examination results, etc. Follow-up examinations were conducted in our outpatient clinic, and all patients continued to receive the prescribed course of treatment after surgical operation. The postoperative follow-up time was not less than 6 months, and all patients underwent a follow-up chest CT scan at approximately 3 to 6 months after the operation, which was compared with the preoperative CT scan.

### Operative procedure

A skin incision was made in the rear outer or front outer intercostal space, and the majority of the rib was resected. The operation then proceeded with the removal of part of the parietal fibrous pleural rind directly below the incision to expose the empyema cavity. Contents were suctioned, and after all of the necrotic material was removed, the chest cavity was washed, and the pus cavity was filled with gauze. The visceral fibrous board was removed from the visceral and parietal pleural folds using a combination of blunt and sharp dissection methods. Then, electrocautery was used to remove the fibrous pleural rind on the surface of the diaphragm, pericardium, and parietal pleura. Finally, the lower lung ligament and pulmonary adhesions around the pus cavity were separated. After all of the fibrous pleural rind was removed, the chest cavity was washed repeatedly, and meticulous hemostasis was obtained. Additionally, 1–2 chest drainage tubes were used to provide sufficient drainage after the surgery.

### Evaluation criteria of therapeutic efficacy

According to the CT imaging changes, the results were evaluated as follows: (1) Cured: full lung expansion, without pleural hypertrophy, and the parietal pleural rind thickness was less than 1 cm; (2) Improved: full lung expansion, and there was no purulent cavity, but the pleural thickness was greater than 1 cm, or a lack of full lung expansion, but there was no liquid cavity left in the chest cavity; (3) Not cured: there was residual purulent cavity, and the lung was partially or not re-expanded compared with that before operation^4^.

### Statistical analysis

Mean, standard deviation (SD), median and interquartile range (IQR) values were used to describe continuous variables. Student's t test was used for comparison of normally distributed continuous variables between groups. Nonnormally distributed continuous variables were compared using the Mann‒Whitney U test. Categorical variables are described as frequencies and percentages. The proportion of categorical variables between the two groups was compared using the x^2^ test, but Fisher’s exact test was used when the sample size was limited. All statistical items were analysed by SPSS23 software. A two-sided α less than 0.05 was considered statistically significant.

## Results

**Figure 1:**
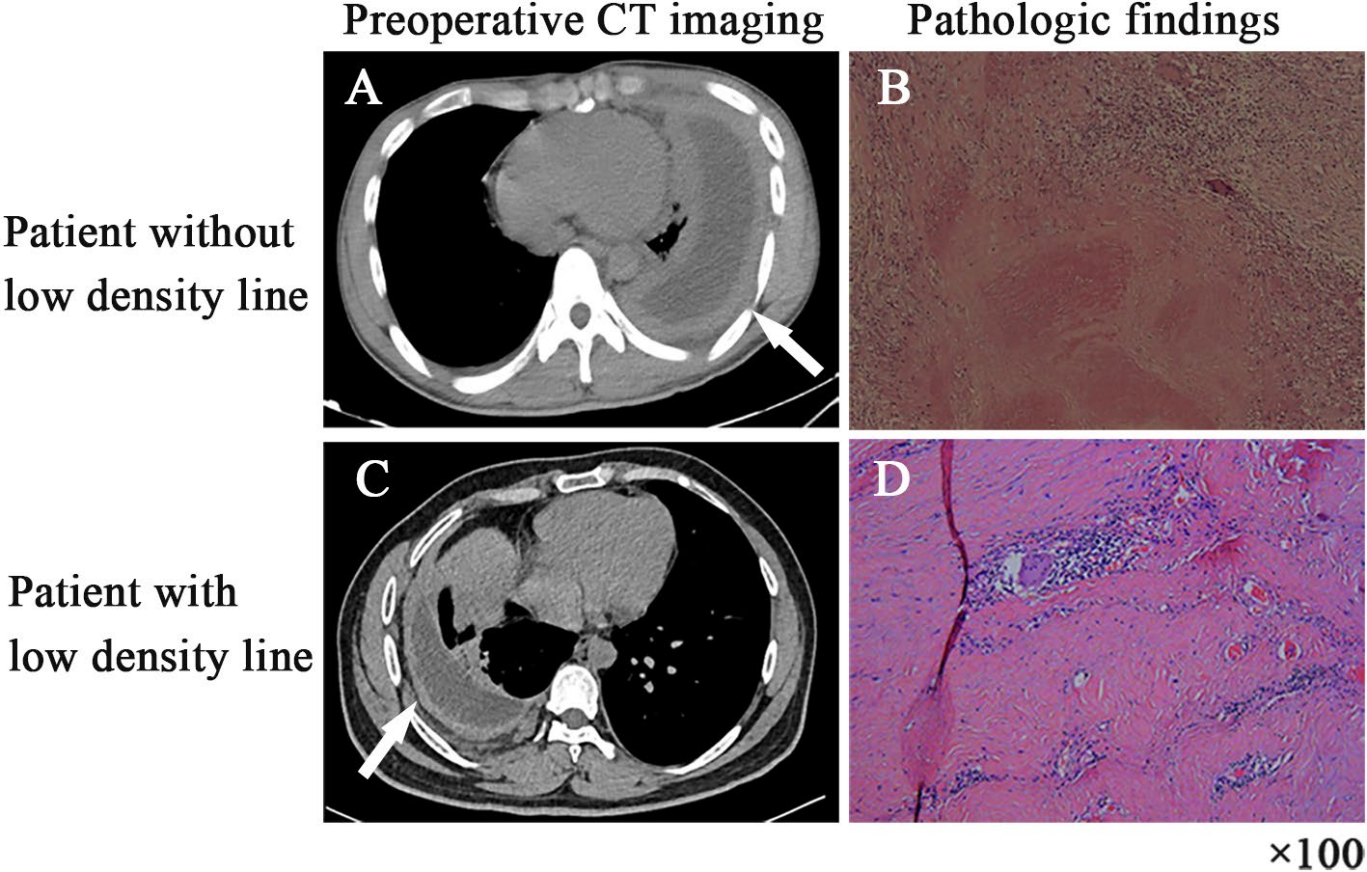
CT and pathological pictures of patients with or without low-density lines (**A**). There was no obvious boundary between the fibrous pleural rind and the parietal pleura (**B**). The typical pathological change in patients without a low-density line was caseous necrosis (**C**). The distinct “low-density line between the fibrous pleural rind and the parietal pleura” (**D**) The typical pathological change in patients with a low-density line was hyperplasia with hyaline degeneration.

A total of 80 patients met the study criteria. Among those, 44 patients had a low-density line between the parietal fibrous pleural rind and chest wall on chest CT scan. Thirty-six patients did not have this imaging finding. The typical manifestations of patients with low-density lines on CT imaging were as follows: the density of intrathoracic lesions was not inhomogeneous, and there was a similar density in the fiberboard and muscle, an obvious gap with the chest wall, and an obvious boundary between the visceral pleura and the parietal pleura. The structure of lesions in the thoracic cavity could be divided into "three layers" (Fig. 1C), and the pathological examination is shown in Fig. 1D. Patients without low-density lines on chest CT are shown in Fig. 1A (pathological report is shown in Fig. 1B).

Detailed demographics are presented in Table 1. There was no significant difference between the two groups in terms of sex, age, ipsilateral pulmonary tuberculosis, narrow rib spaces, side of disease, or positive rate of pathogen detection. The duration of symptoms (*P* = 0.0030) and that of preoperative chemotherapy (*P* = 0.0016) were found to be associated with the appearance of low-density lines on preoperative CT imaging. The erythrocyte sedimentation rate (ESR) of the low-density line group was lower than that of the group without low-density lines, 23 (range 8–57.25) versus42 (range 25–62). Other inflammatory indicators, including C-reactive protein (CRP) (*P* = 0.0027) and leukocyte counts (*P* = 0.0339), were also decreased in the low-density line group. In contrast, the median percentage of lymphocytes in the low-density line group was higher than that in the group without low-density lines, 27.19 (range 21.09–34.48) versus 19.77 (range 15.60–24.10, *P* < 0.0001), and a difference in hemoglobin levels was also observed between the two groups (*P* = 0.0115). The low-density line group had a higher body weight than the group without low-density lines (*P* = 0.0226).

**Table 1:** Preoperative demographic details (n = 80).

Characteristics	Group with low-density lines (n = 44)	Group without low-density lines (n = 36)	*P* value
Sex (n, %)			0.706
Male	38 (86.36)	30 (83.33)	
Female	6 (13.64)	6 (16.67)	
Ipsilateral pulmonary tuberculosis (n, %)			0.651
Yes	31 (70.45)	27 (75.00)	
No	13 (29.55)	9 (25.00)	
Narrow rib spaces (n, %)			0.801
Yes	37 (84.09)	31 (86.11)	
No	7 (15.91)	5 (13.89)	
Side of disease (n, %)			0.964
Left side	21 (47.73)	17 (47.22)	
Right side	23 (52.27)	19 (52.78)	
Aetiological examination (n, %)			0.315
Positive	39 (88.60)	29 (80.60)	
Negative	5 (11.40)	7 (19.40)	
Age, years	31 (24.25, 45)	28 (23, 39.75)	0.2683
Duration of symptoms, m	5.5 (3.625, 11.5)	3 (2, 4.75)	0.003
Duration of chemotherapy, m	4.5 (3, 9)	2 (1.00, 3.75)	0.0016
ESR, mm/h	23 (8, 57.25)	42 (25, 62)	0.0218
CRP, mg/L	15.78 (3.565, 31.88)	26.77 (16.86, 43.04)	0.0027
Leukocyte count, L	5.195 (4.09, 6.10)	6.285 (4.723, 7.778)	0.0339
Lymphocyte proportion, %	27.19 (21.09, 34.48)	19.77 (15.60, 24.10)	< 0.0001
Hemoglobin, g/L	134 (119, 143.8)	119.5 (116, 134.8)	0.0115
Serum albumin, g/L	39.09 ± 3.874	38.55 ± 3.915	0.5378
Body weight, kg	62.42 ± 9.791	57.89 ± 6.952	0.0226

The positive result of aetiological examination indicates that at least one of the following conditions is met: (i) positive for acid-fast bacilli stain; (ii) positive by mycobacterium culture; (iii) PCR assay confirmation of the disease; (iv) GeneXpert MTB/RIF Ultraassay confirmation of the disease.

Compared to patients without low-density lines, those in the low-density line group had a significantly lower operative time (Table 2). The intraoperative blood loss was significantly lower in patients with low-density lines on the CT scan compared with those without low-density lines, i.e., 300 mL (range, 112.5–400 mL) versus 600 mL (range, 362.5–800 mL; *P* < 0.0001). The median volume of catheter drainage 48 h after the operation (*p* = 0.0067) and the median duration of chest tube use (*P* < 0.0001) were both lower in the low-density line group. The median postoperative hospital stay was 13 days (range 11–14.75 days) in the low-density line group and 15 days (range 12–18.50 days) in the group without low-density lines (*P* = 0.0154).

**Table 2:** Intraoperative variables.

Characteristics	Group with low-density lines (n = 44)	Group without low-density lines (n = 36)	*P* value
Operative time, minutes	240.0 (197.5, 303.8)	307.5 (270, 367.5)	0.0003
Intraoperative blood loss, mL	300 (112.5, 400)	600 (362.5, 800)	< 0.0001
Drainage volume during 48 h after operation, mL	790 (642.5, 997.5)	980 (812.5, 1280)	0.0067
Postoperative hospital Stay, d	13 (11, 14.75)	15 (12, 18.50)	0.0154
Duration of chest tube use, d	8 (6, 10)	14 (9, 27)	< 0.0001

The most common pathological feature in both groups was hyperplasia of fibrous connective tissue. A total of 88.64% of participants in the low-density line group showed hyperplasia with hyaline degeneration in pathological examination, while only 41.67% showed hyperplasia with hyaline degeneration in the group without low-density lines (*P* < 0.001). In addition, gaseous necrosis was considerably lower in patients with low-density lines on CT imaging than in those without this imaging finding (*P* = 0.004). Pathological details are described in Table 3.

**Table 3:** Pathological characteristics.

Characteristics	Group with low-density lines (n = 44)	Group without low-density lines (n = 36)	*P* value
Pathological changes (n, %)			
Fibrinoid necrosis	5 (11.36)	2 (5.56)	0.351
Caseous necrosis	25 (56.82)	31 (86.11)	0.004
Granuloma	33 (75.00)	32 (88.89)	0.106
Hyperplasia with hyaline degeneration	39 (88.64)	15 (41.67)	< 0.001
Hyperplasia of fibrous connective tissue	43 (97.73)	34 (94.44)	0.441

**Figure 2:**
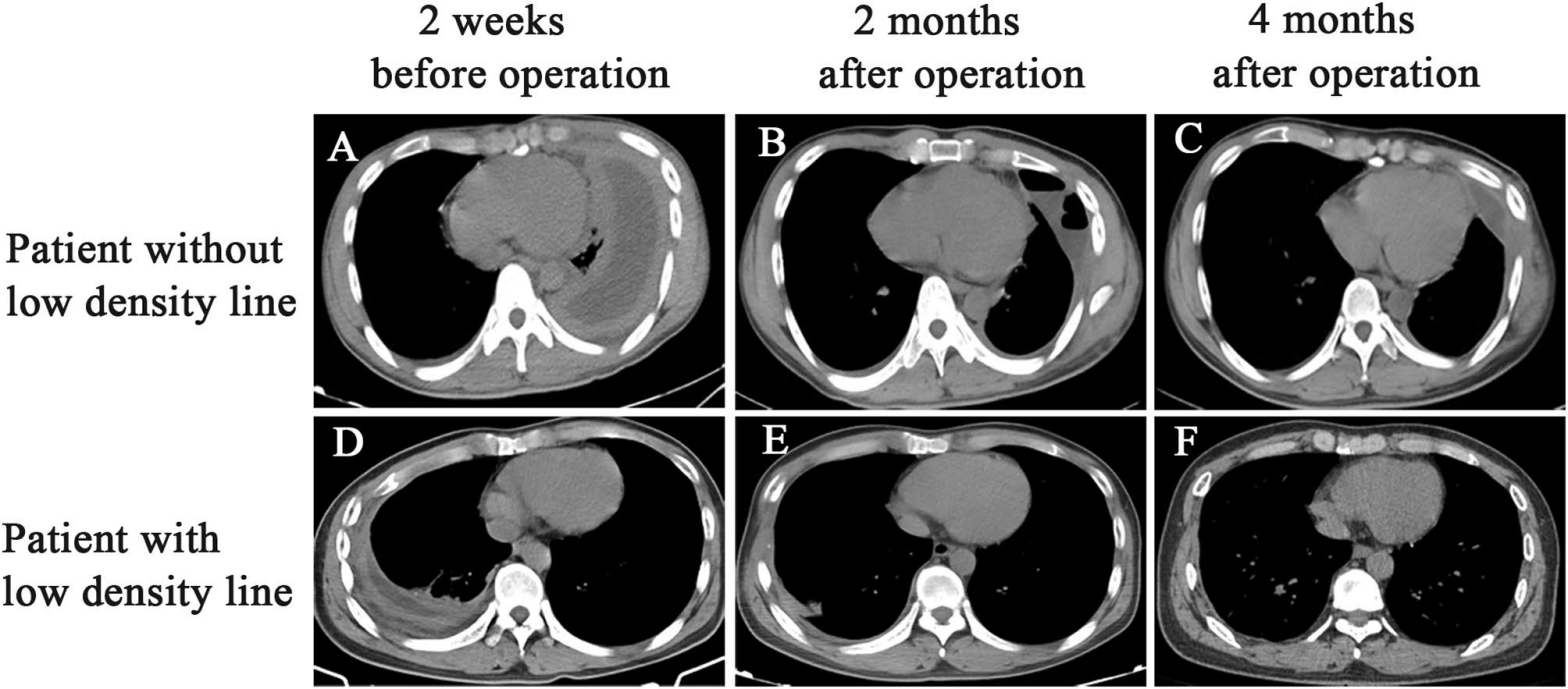
Recovery of the thorax after surgery. Preoperative CT (**A**) showed left empyema, fibrous plate formation, and no obvious low-density line. The operation time was 6 h, the intraoperative bleeding volume was 800 mL, the postoperative exudation volume was 910 mL, and the duration of drainage was 29 days. (**B**,**C**) Are 2 months and 4 months after the operation, respectively. Preoperative CT (**D**) showed right empyema, fibrous plate formation, and a low-density line. The operation lasted 4.5 h, the intraoperative bleeding volume was 300 mL, the postoperative exudation volume was 580 mL, and the duration of drainage was 7 days. (**E**,**F**) were 2 months and 4 months after the operation, respectively.

In addition, preoperative and postoperative chest CT imaging were conducted to evaluate the surgical efficacy. The excellent and good rates were 88.64% in the low-density line group and 63.89% in the group without low-density lines. The surgical outcome of the low-density line group was much better than that of the group without low-density lines (*P* < 0.05) (Table 4). The median follow-up duration was 9 months; there were no deaths in the hospital or at the 6-month of follow-up. Figure 2 shows in detail the characteristics of preoperative and postoperative CT changes in patients with or without low-density lines.

**Table 4:** Comparison of surgical efficacy between the two groups.

Characteristics	Group with low-density lines (n = 44)	Group without low-density lines (n = 36)	*P* value
Surgical efficacy (n, %)			0.042
Lung expansion fully, no pleural hypertrophy or pleural thickness less than 1 cm	39 (88.64)	23 (63.89)	
Lung expansion fully, no residual cavity, but pleural thickness greater than 1 cm	3 (6.82)	4 (11.11)	
Lung expansion not fully, residual fluid free cavity in chest cavity	1 (2.27)	4 (11.11)	
Partial or no re-expansion of the lung, liquid residual cavity	1 (2.27)	5 (13.89)

## Discussion

Tuberculosis is the main cause of empyema in developing countries and is experiencing a reemergence in some Western countries^7–9^. Stage I tuberculous empyema cases are usually cured by an anti-tuberculosis regimen, thoracic drainage, and thoracentesis. In stage II (transitional or fibrinopurulent phase), the current extensive standard is the debridement of the pleural cavity by surgery. Early improper treatment of effusion can lead to many patients progressing to stage III empyema, when surgical intervention will be needed and surgical approaches should be more sophisticated if the empyema is not treated and resolved at this stage^10^. The effect of surgical approaches by decortication of the visceral and parietal pleura in the treatment of stage III tuberculous empyema is gradually becoming recognized and commonly used^6,11^. The optimum timing of surgery is crucial for achieving better outcomes. Nazish Sikander’s group emphasized that preoperative anti-tuberculosis therapy for at least six weeks may yield better outcomes and minimize morbidity^12^. However, some studies have reported a range of two weeks to 12 months of preoperative anti-tuberculous treatment before decortication surgery^13^. Despite numerous studies, there are still considerable differences in the timing of surgery for stage III tuberculous empyema, and it remains controversial^12^. Even if the patients all suffer from chronic tuberculous empyema, their pathological manifestations are inconsistent, making it challenging to determine the optimal operation time by relying only on the preoperative anti-tuberculosis time and the course of disease.

In the early organizing phase, fiberboard is gradually formed due to pleurisy reaction in the visceral and parietal layers, thoracic cellulose, and necrotic deposition to wrap the empyema. The main pathological changes of the fiberboard are caseous necrosis and granuloma, with a low density. Although the fibrous connective tissue proliferated at this time, the fibrous plate remained loose, the inflammatory oedema of the surrounding tissue was aggravated, and the adhesion was still dense. Although CT images can clearly present the fiberboard, the boundary with the surrounding tissue is not clear, forming a "two-layer structure" change and preventing the detection of low-density lines on CT imaging. With the extension of anti-tuberculosis treatment time, the inflammation is gradually controlled (ESR and CRP are decreased). The caseous necrosis with a lower density in the fibrous plate decreases, while the proliferation of fibrous connective tissue and glass-like changes with a higher density increases. The fiberboard density increases gradually, the focus is limited, and the inflammatory oedema of the capsule wall and surrounding tissues gradually subsides. Additionally, long-term atelectasis and other factors jointly lead to narrowing of the costal space and a reduction in the thoracic volume, thereby forming a "three-layer structure", which manifests as a continuous low-density line around the thickened parietal fibrous pleural rind on CT imaging. The density of fat tissue on CT is high during inflammation or oedema, which may be one of the reasons why the low-density lines group had a higher body weight than the group without the low-density lines. In the low-density line group, the boundary between the fiberboard and the visceral pleura was relatively clear, which is conducive to surgical dissection. This difference may explain the significantly reduced operation time, intraoperative blood loss, and postoperative hospital stay of patients in the low-density line group compared with those without low-density lines. It is suggested that the low-density line group in CT staging has a smaller wound, lower risk, faster recovery, and better outcome, and it is therefore recommended that patients without low-density lines but a thick fiberboard should appropriately postpone their operation unless there are special circumstances. In the case of a thin fiberboard and obvious lung recruitment after effective drainage, thoracoscopic-assisted early thoracic dissection can also be considered.

The phenomenon of a "low-density line" was first observed on the CT scan of some patients in 2018, but this phenomenon has not been systematically explained to date. This article comprehensively described the pre, intra- and postoperative conditions of patients with or without low-density lines and emphasized the importance of preoperative CT staging. Preoperative CT staging can reflect the course of disease and the time of preoperative anti-tuberculous therapy. CT scanning is the external manifestation of the pathological changes of empyema. It is more operable than pathological examination. Thus, preoperative CT scanning can be considered a reliable preoperative factor in deciding the surgical management of stage III empyema. However, this study has its own limitations: it only represents stage III tuberculous empyema from a single institute. In this case, the data cannot be generalized. After 2019, open decortication was still one of the preferred procedures in developing countries^6,14^, and video-assisted thoracic surgical decortication technology gradually matured, so all enrolled patients underwent open decortication. These finding provide a foundation for future research in the treatment of tuberculous empyema.

In conclusion, tubercular empyema is common in developing countries, and many patients progressing to stage III need decortication treatment. Reasonable surgical operation timing is crucial for better outcomes and minimizing intraoperative blood loss for patients with stage III tubercular empyema. In the long-term clinical practice of the authors, the appearance of low-density lines on CT imaging indicates that the inflammatory oedema of the fibrous plate and surrounding tissues has subsided, and the fibrous plate forms a clear boundary with visceral pleura, parietal pleura, diaphragm, pericardium and other tissues. Patients with low-density lines on CT imaging are good candidates for open decortication, which is a reliable, and effective therapeutic option for patients with stage III tuberculous empyema who have CT imaging with low-density lines. This approach is worthy of further research.

## Data Availability

The datasets used during the current study available from the corresponding author (Xiyong Dai, daixiyong71@126.com) on reasonable request.
